# Risk prediction of sarcopenia in a large health checkup population: development and validation of a dynamic online nomogram

**DOI:** 10.3389/fpubh.2026.1822652

**Published:** 2026-04-28

**Authors:** Hailin Yang, Cheng Luo, Qian Xiao, Kang Luo, Na Shen

**Affiliations:** 1 Department of Geriatrics, Laboratory of Research and Translation for Geriatric Diseases, The First Affiliated Hospital of Chongqing Medical University, Chongqing, China; 2 Department of Geriatrics, Chongqing Hospital of Traditional Chinese Medicine, Chongqing, China; 3 Department of Critical Care Medicine, The First Affiliated Hospital of Chongqing Medical University, Chongqing, China

**Keywords:** health checkup population, LASSO regression, nomogram, risk prediction model, sarcopenia

## Abstract

**Purpose:**

Sarcopenia is a progressive disorder of skeletal muscle linked to numerous adverse health outcomes. This study aimed to create and validate a nomogram model to predict sarcopenia risk in a large cohort undergoing routine health exams.

**Methods:**

This retrospective study analyzed data derived from standard physical examination indicators collected in a health checkup population. Participants were randomly divided into a training set comprising 70% and a testing set comprising 30%. In the training cohort, key predictors were determined using LASSO regression and subsequent multivariable logistic regression. A predictive nomogram was subsequently constructed. Model performance was assessed through ROC curves, calibration analysis, and decision curve analysis (DCA).

**Results:**

The analysis included 3,277 participants. The final nomogram included eight predictors: sex, calf circumference, body mass index (BMI), employment status, total bilirubin, hemoglobin, total cholesterol and creatinine. A web-based dynamic nomogram was created using this model and is available at https://luokang.shinyapps.io/dynnomapp/. The model exhibited strong discriminative performance, achieving an AUC of 0.909 in the training set and 0.891 in the testing set, demonstrating reliable predictive capability across datasets. The calibration curves indicated a strong correlation between the predicted probabilities and the actual outcomes. Furthermore, decision curve analysis supported the potential clinical utility of the nomogram.

**Conclusion:**

We created and validated a sarcopenia risk prediction model using routinely collected health examination data and transformed it into an accessible online nomogram. The model demonstrates robust predictive capabilities and significant clinical utility, enabling early detection of high-risk sarcopenia cases in health checkup populations.

## 1 Introduction

Sarcopenia is a systemic disorder marked by a gradual reduction in skeletal muscle mass, strength, and physical performance (1, 2). Sarcopenia, a growing global public health issue, is linked to numerous negative outcomes such as falls, fractures, diminished quality of life, elevated hospitalization rates, and increased all-cause mortality (3, 4). Recent studies show an increasing prevalence of sarcopenia among older adults living in communities, closely associated with chronic conditions like diabetes and cardiovascular disease, significantly burdening global healthcare systems (5, 6). With the accelerating pace of population aging, the prevention and management of sarcopenia have become a major focus in contemporary clinical and public health research.

Importantly, accumulating evidence over the past decade suggests that sarcopenia is not confined to older populations (7). Sarcopenia is increasingly affecting younger individuals due to sedentary lifestyles, lack of physical activity, poor dietary habits, and metabolic disorders (8–10). Studies have demonstrated that reductions in muscle mass and functional capacity attributable to secondary causes or lifestyle-related factors are increasingly observed in young and middle-aged adults (11, 12). Failure to identify and intervene in muscle loss during early and midlife may predispose these individuals to a markedly higher risk of frailty and disability later in life. Consequently, shifting the focus from an older adult-centered perspective to a life-course approach is essential for effective health management and disease prevention.

Given its insidious onset and the absence of specific early symptoms, timely screening and risk assessment of sarcopenia in the general population are particularly challenging yet critically important (13, 14). Individuals undergoing routine health examinations represent a large and relatively representative population, offering a valuable opportunity for early disease detection. Currently, sarcopenia diagnosis mainly utilizes dual-energy X-ray absorptiometry (DXA), bioelectrical impedance analysis (BIA), and handgrip strength evaluation (15, 16). However, these methods often require costly equipment and specialized personnel, which substantially limits their feasibility for large-scale screening. There is an urgent requirement to create straightforward, noninvasive, and effective predictive tools to identify individuals at high risk of sarcopenia during health checkups.

Against this backdrop, the present study retrospectively analyzed clinical data from a health examination cohort and developed a dynamic online nomogram to estimate the risk of sarcopenia. This model offers clinicians an accessible tool for early screening and timely intervention, aiming to enhance long-term outcomes and quality of life for at-risk individuals.

## 2 Methods

### 2.1 Study design and participants

This retrospective cross-sectional study analyzed data from individuals who underwent routine health examinations at the First Affiliated Hospital of Chongqing Medical University between November 2017 and November 2019. Eligible participants were adults aged 30–75 years who were able to complete the questionnaire and cooperate with the examination procedures, as this age range covers the majority of adults undergoing routine health examinations.

Participants were excluded if they had limb disabilities or severe psychiatric disorders affecting independent ambulation or anthropometric measurements, serious underlying diseases or malignancies, long-term use of oral glucocorticoids or immunosuppressive agents, or acute infectious conditions at the time of examination, including leukocytosis (white blood cell count > 10 × 10^9^/L) or acute exacerbation of chronic obstructive pulmonary disease. After excluding conditions that could substantially affect body composition, physical function, or laboratory measurements, a total of 3,277 participants were ultimately included in the analysis.

The study adhered to the Declaration of Helsinki and received approval from the Medical Ethics Committee of the First Affiliated Hospital of Chongqing Medical University (Approval No. 2022-K460). This study followed the principles of the Strengthening the Reporting of Observational Studies in Epidemiology (STROBE) guidelines (17) and the Transparent Reporting of a multivariable prediction model for Individual Prognosis or Diagnosis (TRIPOD) statement (18). Participants received comprehensive information about the study objectives and procedures and provided written informed consent before data collection commenced.

### 2.2 Definition of sarcopenia and measurements

Sarcopenia was identified in this study based on the 2025 updated diagnostic criteria of the Asian Working Group for Sarcopenia (AWGS 2025) (19). Participants were identified as having sarcopenia if they exhibited low skeletal muscle mass together with low muscle strength. Recent evidence suggests that sarcopenia is not exclusively confined to older adults, and sarcopenia in younger adults is not uncommon (7). With reference to previous studies, participants aged 30–50 years in the present study were classified using the same age cutoffs as those aged 50–64 years (20).

Appendicular skeletal muscle mass (ASM) was measured using bioelectrical impedance analysis (BIA) with the TANITA MC-780 device (Tanita Corporation, Tokyo, Japan). All measurements were performed under standardized conditions. Participants were instructed to fast prior to assessment, remove shoes and socks to ensure adequate contact between the heels and foot electrodes, grasp the hand electrodes firmly with thumbs placed on the sensors, and maintain an upright posture with both arms fully extended. The appendicular skeletal muscle mass index (ASMI, kg/m^2^) was determined by dividing appendicular skeletal muscle mass (ASM) by the square of the individual’s height. Low muscle mass, as determined by BIA measurements, was defined using age-specific cutoffs: for participants aged 30–64 years, ASMI < 7.6 kg/m^2^ for men and <5.7 kg/m^2^ for women; for participants aged ≥65 years, ASMI <7.0 kg/m^2^ for men and <5.7 kg/m^2^ for women.

Muscle strength was evaluated using handgrip strength (HGS), measured with a handheld dynamometer (CAMRY Model EH101, Guangdong, China) following standardized protocols to ensure accuracy and reproducibility. Participants stood upright with shoulders adducted and neutrally rotated, elbows flexed at 90°, and forearms and wrists in a neutral position, gripping the dynamometer forcefully with thumbs facing upward. Measurements were alternately taken for each hand, and the average of the highest values from both hands was used for further analysis. Low muscle strength was defined using age-specific thresholds: for participants aged 30–64 years, HGS < 34 kg for men and <20 kg for women; for participants aged ≥65 years, HGS < 28 kg for men and <18 kg for women.

### 2.3 Data collection

Comprehensive data were collected from all participants, including demographic characteristics, socioeconomic and lifestyle factors, medical history, anthropometric measurements, vital signs, and laboratory parameters. All questionnaires and physical examinations were administered by trained healthcare professionals following standardized protocols. To ensure data quality and reliability, all collected data were independently entered and verified by two data clerks.

The demographic and socioeconomic variables comprised age (years), sex (male/female), race (Han Chinese/other), educational attainment (less than high school, high school, more than high school), living status (living alone/not), employment status (employed/unemployed), and monthly expenses (less than 1,000, 1,000–3,000, 3,000–6,000, more than 6,000). Lifestyle-related variables comprised spicy food consumption (never, occasionally, often, or every day), yogurt intake (never, occasionally, often, or every day), smoking status (yes or no), alcohol consumption (yes or no), and physical activity level. Physical activity was evaluated via the International Physical Activity Questionnaire (IPAQ) and classified into mild, moderate, or severe levels based on standard scoring guidelines. Medical history variables included self-reported hypertension, diabetes mellitus, and coronary heart disease (CHD), each classified as yes or no. Measurements taken included calf circumference, body mass index (BMI), waist and hip circumferences, as well as systolic and diastolic blood pressure. All measurements were obtained using standardized procedures by trained personnel.

Venous blood samples were collected from all participants after a minimum 8-h fast and analyzed at the clinical laboratory of the First Affiliated Hospital of Chongqing Medical University. Biochemical parameters, including albumin, total bilirubin, *γ*-glutamyl transferase (GGT), alanine aminotransferase (ALT), aspartate aminotransferase (AST), total cholesterol (TC), triglycerides (TG), high-density lipoprotein cholesterol (HDL), low-density lipoprotein cholesterol (LDL), glucose, blood urea nitrogen (BUN), creatinine, and uric acid, were assessed using a fully automated biochemical analyzer (Modular DDP, Roche Diagnostics). Hematological indices, including white blood cell (WBC), red blood cell (RBC), hemoglobin (HB), platelet (PLT), hematocrit (Hct), monocyte, neutrophil, and lymphocyte counts, were measured using an automated hematology analyzer (Boule Medical AB, Sweden).

### 2.4 Model development and evaluation

This study employs cross-sectional data for predictive modeling, with the aim of performing early disease risk assessment based on an individual’s current health status. The dataset was randomly split into a training set and a testing set with a 7:3 ratio. LASSO regression was utilized on the training set to identify potential sarcopenia risk factors in the health checkup population, aiming to minimize covariate multicollinearity and reduce the risk of model overfitting. Ten-fold cross-validation was used to identify the optimal penalty parameter, and variables with non-zero coefficients were retained for inclusion in a multivariable logistic regression model. Effect estimates were quantified using odds ratios (ORs) with corresponding 95% confidence intervals (CIs). Variables with a two-sided *p-*value <0.05 in the multivariable logistic regression model were included in the final predictive model, which was subsequently visualized as a nomogram. An interactive, web-based dynamic nomogram was created using the Shiny framework to support clinical application.

Model performance was evaluated using receiver operating characteristic (ROC) curves and calibration curves. Discriminative ability was assessed using the area under the ROC curve (AUC), which ranges from 0.5 (no discriminative ability) to 1.0 (perfect discrimination). Decision curve analysis (DCA) was performed to evaluate the clinical utility of the model by estimating net benefit across various threshold probabilities.

### 2.5 Statistical analysis

All missing data were below 3%, and missing values were imputed using the k-Nearest Neighbors (KNN) method. Normally distributed continuous variables are reported as mean ± standard deviation (Mean ± SD), while categorical variables are presented as frequencies and percentages [n (%)]. In univariate analyses, categorical variables were assessed using the chi-square test or Fisher’s exact test, and continuous variables were evaluated using Welch’s two-sample *t-*test. A two-sided *p* value < 0.05 was considered statistically significant. All statistical analyses were conducted using R software (version 4.5.1).

## 3 Results

### 3.1 Characteristics of the study population

Table 1 indicates that the study comprised 3,277 participants, with a mean age of 54.60 ± 9.35 years, and 50.69% were male. According to the AWGS 2025 diagnostic criteria, 330 participants (10.07%) were identified as having sarcopenia. A higher occurrence of sarcopenia was observed among participants who were currently employed and those with a history of smoking or alcohol consumption. Participants with sarcopenia also differed significantly in spicy food consumption. Compared with participants without sarcopenia, those with sarcopenia exhibited a lower prevalence of hypertension and significantly reduced measurements in calf circumference, body mass index (BMI), waist and hip circumferences, blood pressure, total bilirubin, liver enzymes (GGT, ALT, and AST), triglycerides, uric acid, creatinine, and lymphocyte count. In contrast, HDL cholesterol levels were notably elevated in the sarcopenia group (all *p* < 0.05). Participants with and without sarcopenia showed no significant differences in age, race, educational attainment, living status, monthly expenses, yogurt intake, physical activity, diabetes mellitus, coronary heart disease, albumin, white blood cell count, red blood cell count, hemoglobin, platelet count, hematocrit, total cholesterol, LDL cholesterol, glucose, blood urea nitrogen, monocyte count, or neutrophil count (all *p* > 0.05). To ensure the robustness and comparability of the datasets, baseline characteristics of participants in the training and testing sets were further described and compared. The results demonstrated no statistically significant differences across any variables between the two cohorts (all *p* > 0.05), indicating good representativeness and consistency of the training and testing datasets (Supplementary Table S1).

**Table 1:** Characteristics of research participants.

Characteristic	Sarcopenia			Statistic^1^	*p*-value
	Overall *N* = 3,277	No *N* = 2,947	Yes *N* = 330		
Age, years	54.60 ± 9.35	54.70 ± 9.44	53.74 ± 8.40	*t* = 1.95	0.052^1^
Sex, *n* (%)				χ^2^ = 134.09	<0.001^2^
Male	1,661 (50.69%)	1,394 (47.30%)	267 (80.91%)		
Female	1,616 (49.31%)	1,553 (52.70%)	63 (19.09%)		
Race, *n* (%)				χ^2^ = 0.10	0.753^2^
Han Chinese	3,196 (97.53%)	2,875 (97.56%)	321 (97.27%)		
Other	81 (2.47%)	72 (2.44%)	9 (2.73%)		
Education, *n* (%)				χ^2^ = 4.90	0.086^2^
<High school	1,314 (40.10%)	1,200 (40.72%)	114 (34.55%)		
High school	990 (30.21%)	884 (30.00%)	106 (32.12%)		
>High school	973 (29.69%)	863 (29.28%)	110 (33.33%)		
Live alone, *n* (%)				χ^2^ = 1.26	0.261^2^
No	3,105 (94.75%)	2,788 (94.60%)	317 (96.06%)		
Yes	172 (5.25%)	159 (5.40%)	13 (3.94%)		
Employment status, *n* (%)				χ^2^ = 31.57	<0.001^2^
No	1,682 (51.33%)	1,561 (52.97%)	121 (36.67%)		
Yes	1,595 (48.67%)	1,386 (47.03%)	209 (63.33%)		
Monthly expenses, *n* (%)				χ^2^ = 7.05	0.070^2^
<1,000	778 (23.74%)	708 (24.02%)	70 (21.21%)		
1,000–3,000	1,493 (45.56%)	1,350 (45.81%)	143 (43.33%)		
3,000–6,000	903 (27.56%)	793 (26.91%)	110 (33.33%)		
>6,000	103 (3.14%)	96 (3.26%)	7 (2.12%)		
Spicy food, *n* (%)				χ^2^ = 9.22	0.026^2^
Never	358 (10.92%)	328 (11.13%)	30 (9.09%)		
Occasionally	1,597 (48.73%)	1,444 (49.00%)	153 (46.36%)		
Often	1,033 (31.52%)	907 (30.78%)	126 (38.18%)		
Every day	289 (8.82%)	268 (9.09%)	21 (6.36%)		
Yogurt intake, *n* (%)				χ^2^ = 5.15	0.161^2^
Never	1,505 (45.93%)	1,334 (45.27%)	171 (51.82%)		
Occasionally	1,465 (44.71%)	1,334 (45.27%)	131 (39.70%)		
Often	250 (7.63%)	227 (7.70%)	23 (6.97%)		
Every day	57 (1.74%)	52 (1.76%)	5 (1.52%)		
Smoke, *n* (%)				χ^2^ = 58.14	<0.001^2^
No	2,774 (84.65%)	2,542 (86.26%)	232 (70.30%)		
Yes	503 (15.35%)	405 (13.74%)	98 (29.70%)		
Drink, *n* (%)				χ^2^ = 51.67	<0.001^2^
No	2,475 (75.53%)	2,279 (77.33%)	196 (59.39%)		
Yes	802 (24.47%)	668 (22.67%)	134 (40.61%)		
Physical activity, *n* (%)				χ^2^ = 2.00	0.367^2^
Mild	513 (15.65%)	468 (15.88%)	45 (13.64%)		
Moderate	2,032 (62.01%)	1,829 (62.06%)	203 (61.52%)		
Severe	732 (22.34%)	650 (22.06%)	82 (24.85%)		
Hypertension, *n* (%)				χ^2^ = 11.15	<0.001^2^
No	2,972 (90.69%)	2,656 (90.13%)	316 (95.76%)		
Yes	305 (9.31%)	291 (9.87%)	14 (4.24%)		
Diabetes, *n* (%)				χ^2^ = 1.61	0.204^2^
No	3,193 (97.44%)	2,868 (97.32%)	325 (98.48%)		
Yes	84 (2.56%)	79 (2.68%)	5 (1.52%)		
CHD, *n* (%)					0.106^3^
No	3,249 (99.15%)	2,919 (99.05%)	330 (100.00%)		
Yes	28 (0.85%)	28 (0.95%)	0 (0.00%)		
Calf circumference, cm	34.36 ± 2.73	34.49 ± 2.72	33.19 ± 2.48	*t* = 8.97	<0.001^1^
BMI, kg/m^2^	24.07 ± 3.03	24.39 ± 2.92	21.20 ± 2.44	*t* = 22.04	<0.001^1^
Waist, cm	82.99 ± 9.13	83.65 ± 9.08	77.09 ± 7.28	*t* = 15.09	<0.001^1^
Hip circumference, cm	95.39 ± 5.39	95.79 ± 5.35	91.83 ± 4.40	*t* = 15.14	<0.001^1^
Systolic pressure, mmHg	128.49 ± 18.93	129.24 ± 18.93	121.84 ± 17.58	*t* = 7.20	<0.001^1^
Diastolic pressure, mmHg	78.06 ± 11.50	78.44 ± 11.57	74.63 ± 10.25	*t* = 6.32	<0.001^1^
Albumin, g/L	47.13 ± 2.86	47.11 ± 2.83	47.30 ± 3.18	*t* = −1.06	0.290^1^
Total bilirubin, μmol/L	12.81 ± 5.14	12.87 ± 5.19	12.25 ± 4.69	*t* = 2.25	0.025^1^
GGT, U/L	34.05 ± 42.38	34.65 ± 43.38	28.76 ± 31.71	*t* = 3.07	0.002^1^
ALT, U/L	24.84 ± 18.01	25.30 ± 18.47	20.71 ± 12.52	*t* = 5.97	<0.001^1^
AST, U/L	23.91 ± 11.28	24.06 ± 11.50	22.55 ± 9.05	*t* = 2.79	0.006^1^
WBC, ×10^9^/L	6.00 ± 1.35	6.01 ± 1.34	5.92 ± 1.45	*t* = 1.07	0.285^1^
RBC, ×10^12^/L	4.76 ± 0.50	4.77 ± 0.50	4.74 ± 0.50	*t* = 0.82	0.411^1^
HB, g/L	145.72 ± 15.55	145.79 ± 15.63	145.09 ± 14.82	*t* = 0.80	0.422^1^
PLT, ×10^9^/L	204.93 ± 56.13	204.71 ± 55.99	206.92 ± 57.41	*t* = −0.67	0.506^1^
Hct, %	43.78 ± 4.10	43.79 ± 4.12	43.72 ± 3.97	*t* = 0.30	0.766^1^
TC, mmol/L	5.10 ± 0.94	5.09 ± 0.95	5.14 ± 0.88	*t* = −0.98	0.326^1^
TG, mmol/L	1.75 ± 1.45	1.77 ± 1.48	1.56 ± 1.10	*t* = 3.16	0.002^1^
HDL, mmol/L	1.44 ± 0.36	1.42 ± 0.35	1.54 ± 0.36	*t* = −5.77	<0.001^1^
LDL, mmol/L	3.22 ± 0.84	3.22 ± 0.84	3.20 ± 0.81	*t* = 0.37	0.708^1^
Glucose, mmol/L	5.81 ± 1.57	5.82 ± 1.57	5.65 ± 1.63	*t* = 1.84	0.066^1^
BUN, mmol/L	5.51 ± 1.41	5.52 ± 1.39	5.38 ± 1.51	*t* = 1.64	0.102^1^
Creatinine, mmol/L	68.91 ± 16.19	69.09 ± 16.38	67.30 ± 14.25	*t* = 2.14	0.033^1^
Uric acid, mmol/L	337.53 ± 87.86	339.34 ± 87.93	321.41 ± 85.67	*t* = 3.59	<0.001^1^
Monocyte, ×10^9^/L	0.38 ± 0.15	0.38 ± 0.15	0.39 ± 0.15	*t* = −0.77	0.441^1^
Neutrophil, ×10^9^/L	3.45 ± 1.03	3.45 ± 1.01	3.50 ± 1.15	*t* = −0.83	0.405^1^
Lymphocyte, ×10^9^/L	1.96 ± 0.56	1.97 ± 0.56	1.84 ± 0.54	*t* = 4.01	<0.001^1^

^1^Welch Two Sample *t*-test. ^2^Pearson’s Chi-squared test. ^3^Fisher’s exact test. SD (standard deviation), CHD (coronary heart disease), BMI (body mass index), ALT (alanine aminotransferase), AST (aspartate aminotransferase), WBC (white blood cell count), RBC (red blood cell count), HB (hemoglobin), PLT (platelet count), Hct (hematocrit), TC (total cholesterol), TG (triglycerides), HDL (high-density lipoprotein), LDL (low-density lipoprotein), BUN (blood urea nitrogen).

### 3.2 Development and visualization of predictive variables

**Figure 1:**
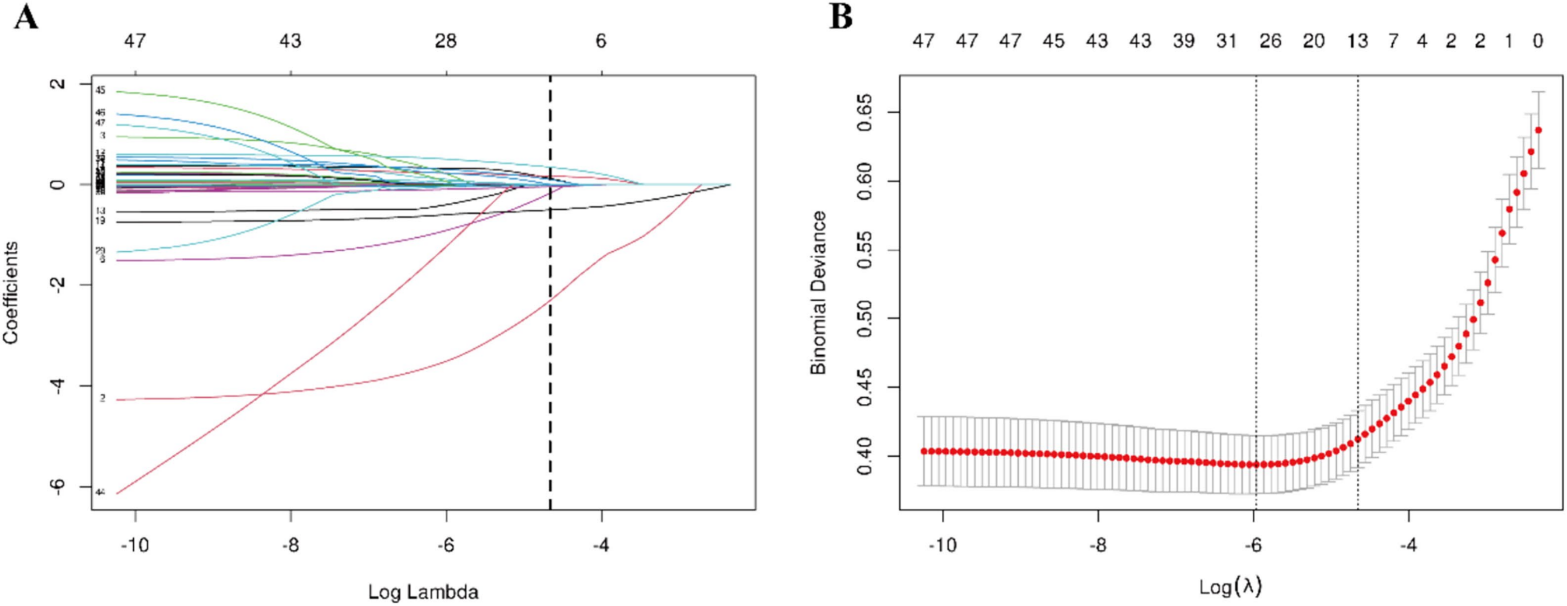
Employing LASSO regression for feature selection. **(A)** Coefficient profiles of the 49 candidate variables under varying levels of regularization. The *x*-axis represents the log-transformed penalty parameter (*λ*), and the *y*-axis denotes the corresponding regression coefficients. As λ increases, penalization is strengthened, progressively shrinking coefficients toward zero; variables with coefficients reduced to zero are excluded from the model. **(B)** Ten-fold cross-validation was performed to evaluate model performance across different λ values using binomial deviance as the criterion. The red dots indicate the mean cross-validated deviance, with vertical bars representing the corresponding 95% confidence intervals. The dashed vertical line denotes the optimal λ that achieved the best model performance and was used to determine the predictors retained in the final model.

**Figure 2:**
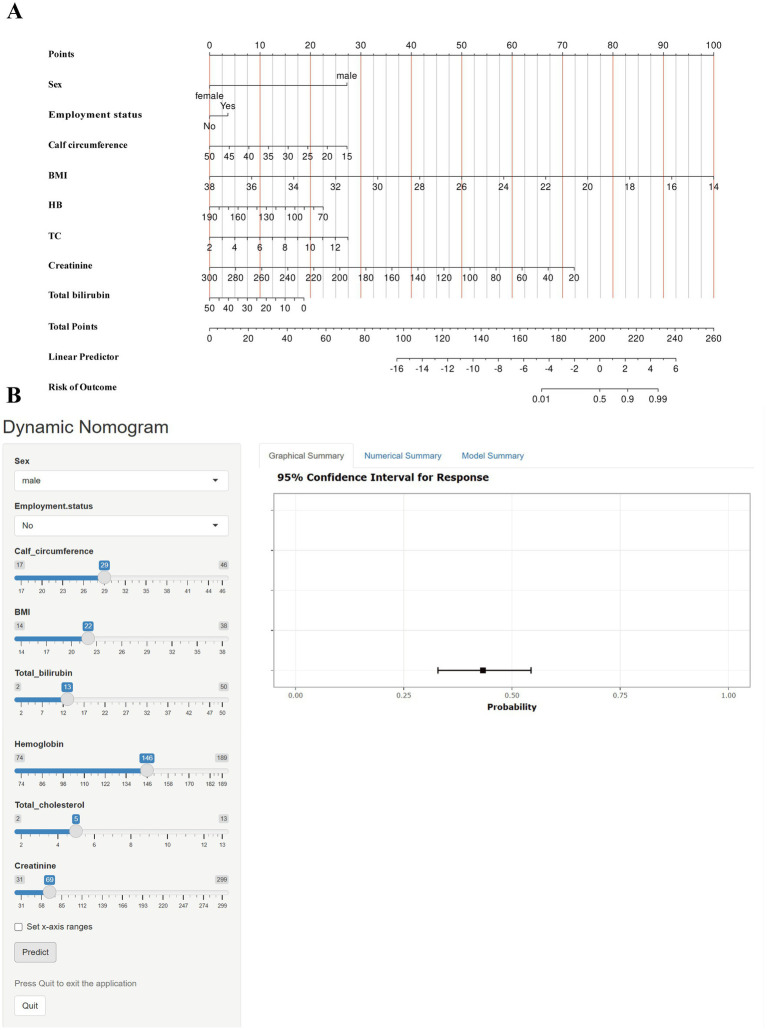
Development and visualization of the sarcopenia risk prediction tool. **(A)** A nomogram constructed based on LASSO regression and multivariable logistic regression for estimating individualized risk of sarcopenia in the health checkup population. For each predictor, users can locate the corresponding value on the scale to obtain a score; the total score is then summed to derive the predicted probability of sarcopenia. **(B)** Interface of the web-based dynamic nomogram. Clinicians can use the online application at https://luokang.shinyapps.io/dynnomapp/ to enter patient-specific variables and instantly receive the model-estimated sarcopenia risk by clicking the “Predict” button. To ensure proper system operation, users are advised to click the “Exit” button to terminate the session after use.

LASSO regression was initially employed in the training set to identify essential predictors of sarcopenia, resulting in the selection of 13 candidate variables: sex, body mass index (BMI), living alone, calf circumference, total bilirubin, creatinine, hemoglobin (HB), systolic pressure, alanine aminotransferase (ALT), total cholesterol (TC), employment status, smoking, and drinking (Figure 1 and Supplementary Figure S1). These variables were then entered simultaneously into a multivariable logistic regression model to assess their independent predictive value. Predictors that remained statistically significant were retained and incorporated into the final model (Supplementary Table S2). The discriminatory performance of the final model was further compared with that of a simplified model including only sex and BMI using the DeLong test. The final model showed a significantly higher AUC than the simplified model (0.916 [0.898–0.934] vs. 0.892 [0.870–0.913], *p* < 0.001; Supplementary Figure S2). Based on the results of these analyses, a nomogram comprising eight independent predictors—sex, calf circumference, body mass index (BMI), employment status, total bilirubin, hemoglobin, total cholesterol, and creatinine—was developed to estimate the risk of sarcopenia in the health checkup population (Figure 2A). In the nomogram, each predictor was assigned a corresponding score, and the total score was summed to determine an individual’s predicted probability of sarcopenia. To enhance clinical accessibility and practical applicability, a web-based dynamic nomogram was additionally developed (Figure 2B) and is publicly available at https://luokang.shinyapps.io/dynnomapp/. This interactive visualization tool offers healthcare professionals an intuitive approach for personalized risk assessment, facilitating early detection and prevention of sarcopenia during routine health checkups.

### 3.3 Evaluation of the nomogram

**Figure 3:**
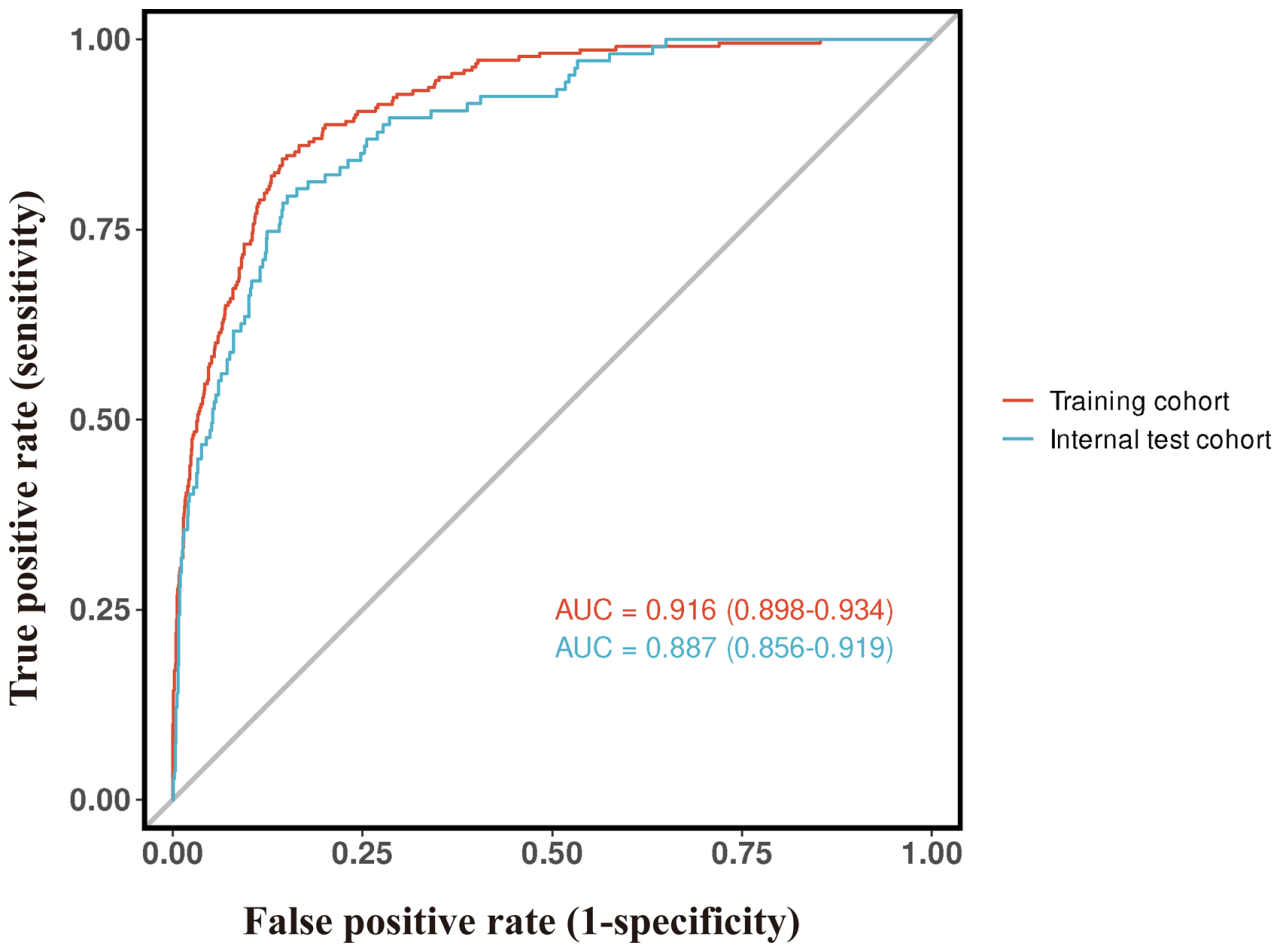
ROC curve of the prediction model.

**Figure 4:**
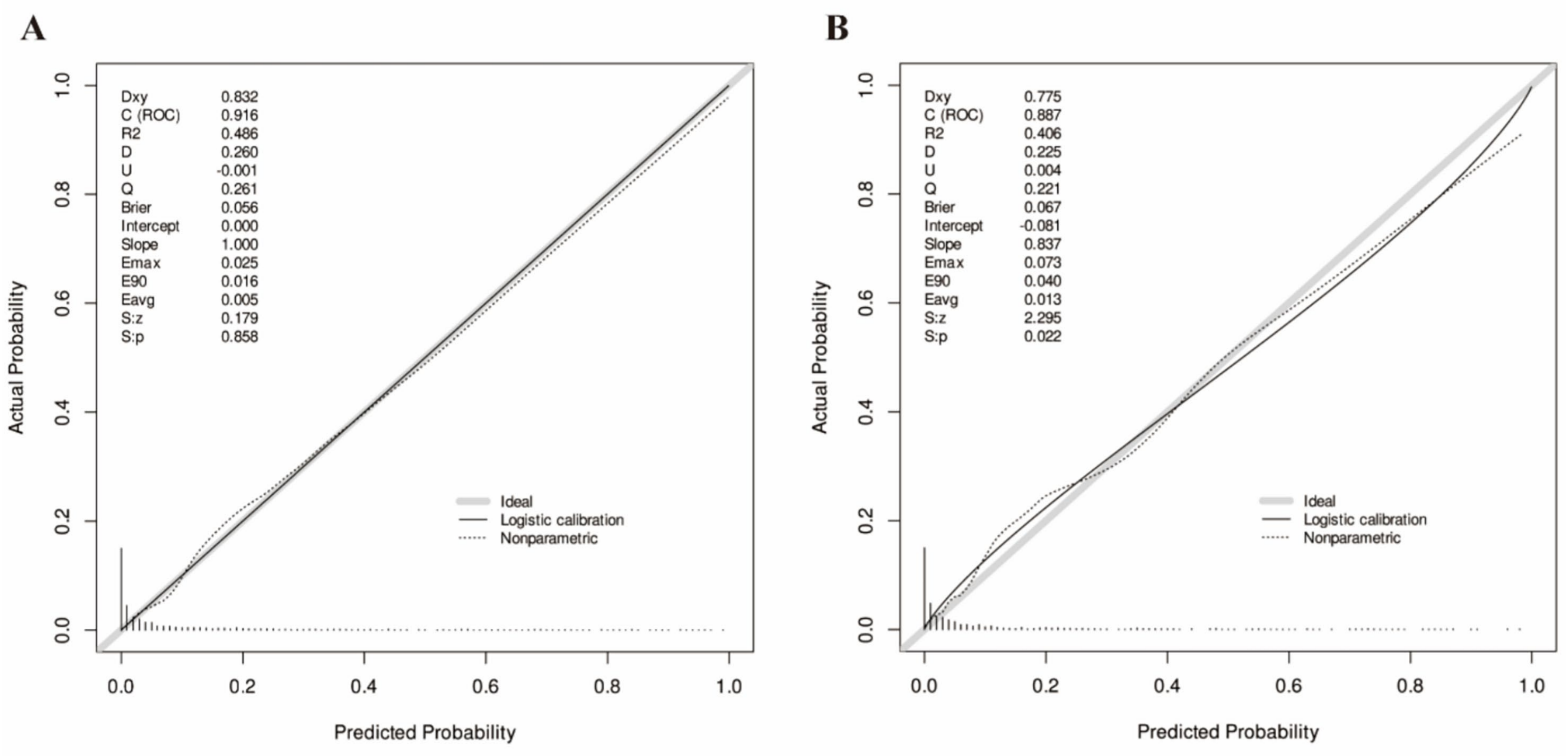
Calibration curves for the training set **(A)** and the testing set **(B)**.

**Figure 5:**
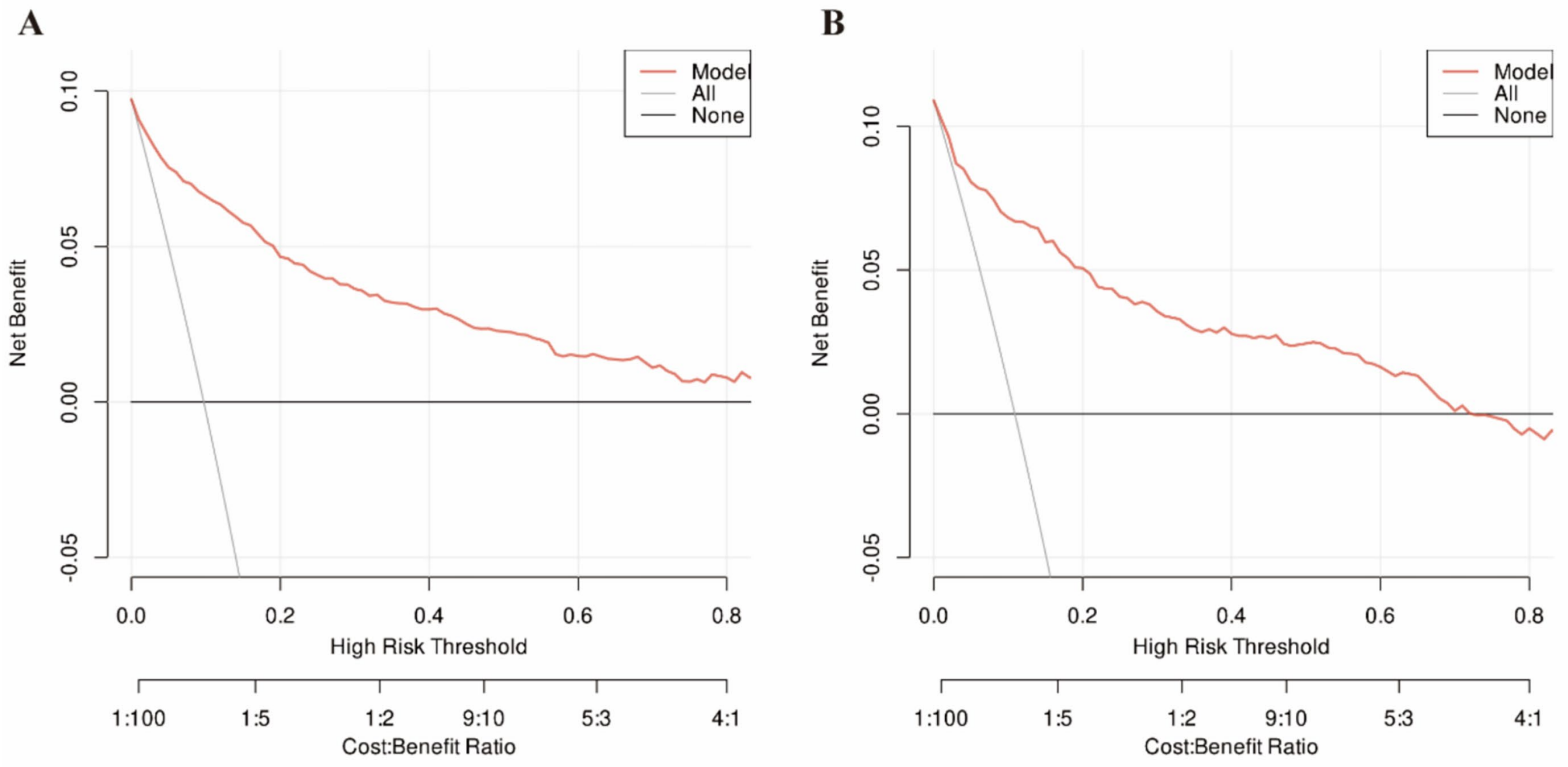
Decision curves for training set **(A)** and testing set **(B)**.

The nomogram’s discriminative ability was evaluated through receiver operating characteristic (ROC) curve analysis. Figure 3 demonstrates the model’s robust discrimination, achieving an AUC of 0.916 (95% CI: 0.898–0.934) in the training set and maintaining consistent performance in the independent testing set with an AUC of 0.887 (95% CI: 0.856–0.919). The calibration of the nomogram was further evaluated using calibration curves (Figure 4). In both the training and testing sets, the predicted probabilities were generally close to the observed probabilities, and the calibration curves were in good agreement with the ideal reference line, suggesting satisfactory consistency between predicted risk and actual outcomes. The clinical utility of the nomogram was assessed by decision curve analysis (DCA) (Figure 5). The DCA curves showed that the nomogram provided a greater net benefit than the treat-all or treat-none strategies across a range of threshold probabilities in both datasets, indicating that the model may be useful in supporting clinical decision-making for sarcopenia risk assessment in the health checkup population. Overall, these findings suggest that the proposed nomogram demonstrates strong discriminative ability, reliable calibration, and potential clinical utility for predicting sarcopenia risk in health checkup populations.

## 4 Discussion

This study created and validated a dynamic, web-based nomogram to predict sarcopenia risk using data from a large health checkup cohort. The model demonstrated strong discriminative ability, good calibration, and favorable clinical net benefit, supporting its robustness and practical relevance. The final model incorporated eight independent predictors: sex, calf circumference, body mass index (BMI), employment status, total bilirubin, hemoglobin, total cholesterol and creatinine. These variables are routinely collected during standard health examinations, highlighting the ease of implementation and clinical applicability of the proposed tool. The nomogram demonstrated excellent discriminative ability and stable predictive performance, achieving AUCs of 0.916 in the training set and 0.887 in the validation set. Decision curve analysis indicated that the model offers significant net benefits across various threshold probabilities, highlighting its potential utility for sarcopenia screening and risk stratification in health checkup populations.

Our findings corroborate previous research by identifying calf circumference and body mass index (BMI) as significant inverse predictors of sarcopenia (21, 22). Calf circumference, a straightforward anthropometric measure indicative of appendicular muscle mass, is strongly correlated with skeletal muscle mass and serves as an effective surrogate marker for community-based sarcopenia screening (23). A lower BMI may indicate insufficient muscle mass and reduced nutritional reserves, thereby increasing susceptibility to sarcopenia (24). Previous studies indicate that individuals with a BMI of 18.5–23.9 kg/m^2^ have a 5.16-fold higher risk of sarcopenia, while those with a BMI below 18.5 kg/m^2^ have a 6.06-fold higher risk compared with individuals in higher BMI categories (25). In addition to the anthropometric indicators, working status was also identified as a risk factor for sarcopenia in our predictive model. This finding may be related to the occupational characteristics of the study population. A considerable proportion of individuals undergoing regular health examinations are office workers, whose occupations are typically characterized by prolonged sedentary behavior and low levels of physical activity. Previous studies have consistently demonstrated that sedentary behavior is significantly associated with an increased risk of sarcopenia (26). Therefore, working status may, to some extent, reflect a sedentary lifestyle pattern, thereby contributing to the risk of sarcopenia.

In addition, this study found that individuals with sarcopenia exhibited lower levels of total bilirubin, hemoglobin, and creatinine, suggesting that oxidative stress status, nutritional condition, and skeletal muscle–related metabolic indicators are closely associated with declines in muscle mass. Higher total bilirubin levels may reflect enhanced antioxidant capacity and have been associated with a reduced risk of sarcopenia (27). Reduced hemoglobin concentrations often indicate poor nutritional status or an increased burden of chronic disease, both of which can adversely affect the maintenance of skeletal muscle function (28). Serum creatinine, as a terminal product of muscle metabolism, serves as an indirect marker of skeletal muscle mass (29); lower creatinine levels typically reflect reduced muscle mass. This observation is consistent with previous findings demonstrating a strong association between muscle-derived metabolic markers and sarcopenia (30, 31). Collectively, these biological pathways—spanning metabolic, nutritional, and inflammatory dimensions—provide mechanistic support for the validity of the proposed predictive model. Our study identified total cholesterol (TC) as a risk factor for sarcopenia. Previous studies have demonstrated a significant association between dyslipidemia and sarcopenia (32, 33). Elevated TC levels may reflect underlying lipid metabolic dysfunction and may further be linked to pathological processes such as chronic low-grade inflammation, insulin resistance, and deterioration in muscle quality (31, 34). We observed a higher prevalence of sarcopenia in men in the present study. However, previous studies have reported inconsistent findings regarding sex differences in sarcopenia prevalence, with some studies in older Chinese populations showing a higher prevalence in women than in men (35). This discrepancy may be related to differences in study populations, as our study was based on a health checkup population aged 30–75 years rather than predominantly older or clinically vulnerable populations. Therefore, the observed association should be interpreted cautiously.

Unlike earlier models focused on specific clinical groups such as patients with diabetes, non–small cell lung cancer, or middle-aged and older populations (36–39), the predictive model developed in this study was designed for a broader health checkup population, which may enhance its screening utility and practical applicability. By relying exclusively on routinely collected examination parameters, our model may be more feasible and scalable in primary healthcare settings and health examination centers. A closely related study by Chong et al. developed and externally validated a machine learning model for predicting sarcopenia in Korean middle-aged adults (39). Notably, several similarities were observed between the two studies. First, sex was retained in both models, supporting the importance of sex-related heterogeneity in sarcopenia prediction, although the observed patterns differed. Chong et al. reported a higher prevalence of sarcopenia in women, whereas our study showed a higher prevalence in men. Second, both studies highlighted body habitus as an important predictor. Chong et al. found that lower BMI, rather than obesity, was associated with a higher risk of sarcopenia, which is broadly consistent with the inclusion of BMI in our final model. Third, both studies retained lipid-related indicators, although these were defined differently, with hypercholesterolemia in Chong et al. and total cholesterol in our model. In contrast, the remaining predictors differed substantially. Chong et al. emphasized factors such as age, protein intake, drinking, and subjective health perception, whereas our final nomogram additionally included calf circumference, employment status, total bilirubin, hemoglobin, and creatinine. These differences may reflect variations in the target population, available candidate variables, and modeling strategy. Specifically, Chong et al. focused on a nationally representative middle-aged population and compared multiple machine learning algorithms with external validation, whereas our study targeted a broader health checkup population aged 30–75 years and emphasized a regression-based nomogram using routinely available examination variables. Therefore, the two models provide partially consistent evidence, and further multicenter studies are needed to validate and extend these findings. Moreover, the development of a dynamic web-based nomogram using the R Shiny framework enables real-time estimation of sarcopenia risk at the individual level. This tool may help clinicians rapidly identify high-risk individuals during routine health examinations and facilitate timely implementation of personalized nutritional support, targeted exercise interventions, and appropriate follow-up strategies, which may contribute to the early prevention and management of sarcopenia.

Although this study benefits from a large sample size, rigorous modeling procedures, and strong model performance, several limitations should be acknowledged. First, the cross-sectional design precludes causal inference; therefore, prospective longitudinal studies are warranted to further examine the temporal sensitivity of the identified predictors and their roles in the progression of sarcopenia. Second, the data were derived from a single center, which may introduce regional bias and limit the generalizability of the model. In addition, the applicability of the model to broader age groups remains unclear. Future multicenter studies including participants with broader age ranges are needed for external validation. Third, the predictive variables in this study were primarily limited to routine health examination measures, and more comprehensive biomarkers or advanced modalities such as imaging-based metrics were not included. Integrating multidimensional data in future research may enable the development of more precise sarcopenia prediction models and further enhance discriminative performance and clinical applicability. In addition, as this study focused on sarcopenia rather than sarcopenic obesity, the proposed model may have limited ability to specifically identify this clinically important phenotype.

## 5 Conclusion

This study created an interactive, web-based nomogram to assess sarcopenia risk in a large health checkup cohort. The model exhibited outstanding discriminative ability, reliable calibration, and positive clinical net benefit in both the training and testing datasets, offering healthcare professionals a convenient and precise risk assessment tool. This tool can aid in the early detection of individuals at high risk for sarcopenia, supporting personalized nutritional and exercise interventions to enhance health outcomes. Further research using multicenter data and longitudinal validation is necessary to improve the model’s generalizability and clinical relevance.

## Data Availability

The raw data supporting the conclusions of this article will be made available by the authors, without undue reservation. The code used for statistical analysis and model development is publicly available on GitHub at: https://github.com/shanzhatangyuan/predictive_model.
