# Supplementary material for: Risk prediction of sarcopenia in a large health checkup population: development and validation of a dynamic online nomogram
Source: Front Public Health. 2026 Apr 28;14:1822652. doi: 10.3389/fpubh.2026.1822652 (PMC13160820; doi:10.3389/fpubh.2026.1822652)
Supplement: Supplementary file 1 [file Table_1.DOCX]

**Table S1.** Baseline characteristics of the training and validation sets.

| **Characteristic** | **DATA** | | | **p-value** |
| --- | --- | --- | --- | --- |
|  | **Overall  N = 3,277** | **Training set  N = 2,294** | **Testing set  N = 983** |  |
| **Age, Mean ± SD** | 54.60 ± 9.35 | 54.76 ± 9.34 | 54.23 ± 9.36 | 0.133^1^ |
| **Sex, n (%)** |  |  |  | 0.834^2^ |
| Male | 1,661 (50.7%) | 1,160 (50.6%) | 501 (51.0%) |  |
| Female | 1,616 (49.3%) | 1,134 (49.4%) | 482 (49.0%) |  |
| **Race, n (%)** |  |  |  | 0.100^2^ |
| Han Chinese | 3,196 (97.53%) | 2,244 (97.82%) | 952 (96.85%) |  |
| Other | 81 (2.47%) | 50 (2.18%) | 31 (3.15%) |  |
| **Education, n (%)** |  |  |  | 0.706^2^ |
| ˂ High school | 1,314 (40.10%) | 925 (40.32%) | 389 (39.57%) |  |
| High school | 990 (30.21%) | 683 (29.77%) | 307 (31.23%) |  |
| ˃ High school | 973 (29.69%) | 686 (29.90%) | 287 (29.20%) |  |
| **Live alone, n (%)** |  |  |  | 0.945^2^ |
| No | 3,105 (94.75%) | 2,174 (94.77%) | 931 (94.71%) |  |
| Yes | 172 (5.25%) | 120 (5.23%) | 52 (5.29%) |  |
| **Employment status, n (%)** |  |  |  | 0.207^2^ |
| No | 1,682 (51.33%) | 1,194 (52.05%) | 488 (49.64%) |  |
| Yes | 1,595 (48.67%) | 1,100 (47.95%) | 495 (50.36%) |  |
| **Monthly expenses, n (%)** |  |  |  | 0.567^2^ |
| ˂ 1000 | 778 (23.74%) | 559 (24.37%) | 219 (22.28%) |  |
| 1000-3000 | 1,493 (45.56%) | 1,032 (44.99%) | 461 (46.90%) |  |
| 3000-6000 | 903 (27.56%) | 633 (27.59%) | 270 (27.47%) |  |
| ˃ 6000 | 103 (3.14%) | 70 (3.05%) | 33 (3.36%) |  |
| **Spicy food, n (%)** |  |  |  | 0.070^2^ |
| Never | 358 (10.92%) | 236 (10.29%) | 122 (12.41%) |  |
| Occasionally | 1,597 (48.73%) | 1,110 (48.39%) | 487 (49.54%) |  |
| Often | 1,033 (31.52%) | 751 (32.74%) | 282 (28.69%) |  |
| Every day | 289 (8.82%) | 197 (8.59%) | 92 (9.36%) |  |
| **Yogurt intake, n (%)** |  |  |  | 0.921^2^ |
| Never | 1,505 (45.93%) | 1,049 (45.73%) | 456 (46.39%) |  |
| Occasionally | 1,465 (44.71%) | 1,029 (44.86%) | 436 (44.35%) |  |
| Often | 250 (7.63%) | 174 (7.59%) | 76 (7.73%) |  |
| Every day | 57 (1.74%) | 42 (1.83%) | 15 (1.53%) |  |
| **Drink, n (%)** |  |  |  | 0.959^2^ |
| No | 2,475 (75.53%) | 1,732 (75.50%) | 743 (75.58%) |  |
| Yes | 802 (24.47%) | 562 (24.50%) | 240 (24.42%) |  |
| **Smoke, n (%)** |  |  |  | 0.605^2^ |
| No | 2,774 (84.65%) | 1,937 (84.44%) | 837 (85.15%) |  |
| Yes | 503 (15.35%) | 357 (15.56%) | 146 (14.85%) |  |
| **Physical activity, n (%)** |  |  |  | 0.696^2^ |
| Mild | 513 (15.65%) | 353 (15.39%) | 160 (16.28%) |  |
| Moderate | 2,032 (62.01%) | 1,421 (61.94%) | 611 (62.16%) |  |
| Severe | 732 (22.34%) | 520 (22.67%) | 212 (21.57%) |  |
| **Hypertension, n (%)** |  |  |  | 0.265^2^ |
| No | 2,972 (90.69%) | 2,072 (90.32%) | 900 (91.56%) |  |
| Yes | 305 (9.31%) | 222 (9.68%) | 83 (8.44%) |  |
| **Diabetes, n (%)** |  |  |  | 0.311^2^ |
| No | 3,193 (97.44%) | 2,231 (97.25%) | 962 (97.86%) |  |
| Yes | 84 (2.56%) | 63 (2.75%) | 21 (2.14%) |  |
| **CHD, n (%)** |  |  |  | 0.562^2^ |
| No | 3,249 (99.15%) | 2,273 (99.08%) | 976 (99.29%) |  |
| Yes | 28 (0.85%) | 21 (0.92%) | 7 (0.71%) |  |
| **Calf circumference, Mean ± SD** | 34.36 ± 2.73 | 34.34 ± 2.72 | 34.41 ± 2.74 | 0.462^1^ |
| **BMI, Mean ± SD** | 24.07 ± 3.03 | 24.09 ± 3.04 | 24.01 ± 3.00 | 0.532^1^ |
| **Waist, Mean ± SD** | 82.99 ± 9.13 | 83.01 ± 9.20 | 82.94 ± 8.99 | 0.858^1^ |
| **Hip circumference, Mean ± SD** | 95.39 ± 5.39 | 95.41 ± 5.43 | 95.36 ± 5.30 | 0.822^1^ |
| **Systolic pressure, Mean ± SD** | 128.49 ± 18.93 | 128.83 ± 18.87 | 127.70 ± 19.07 | 0.118^1^ |
| **Diastolic pressure, Mean ± SD** | 78.06 ± 11.50 | 78.22 ± 11.51 | 77.68 ± 11.46 | 0.221^1^ |
| **Albumin, Mean ± SD** | 47.13 ± 2.86 | 47.14 ± 2.86 | 47.12 ± 2.88 | 0.853^1^ |
| **Total bilirubin, Mean ± SD** | 12.81 ± 5.14 | 12.78 ± 5.10 | 12.88 ± 5.24 | 0.609^1^ |
| **GGT, Mean ± SD** | 34.05 ± 42.38 | 33.75 ± 40.45 | 34.76 ± 46.58 | 0.556^1^ |
| **ALT, Mean ± SD** | 24.84 ± 18.01 | 24.92 ± 18.41 | 24.63 ± 17.05 | 0.660^1^ |
| **AST, Mean ± SD** | 23.91 ± 11.28 | 23.86 ± 10.55 | 24.02 ± 12.85 | 0.739^1^ |
| **WBC, Mean ± SD** | 6.00 ± 1.35 | 6.02 ± 1.37 | 5.95 ± 1.30 | 0.178^1^ |
| **RBC, Mean ± SD** | 4.76 ± 0.50 | 4.76 ± 0.50 | 4.77 ± 0.51 | 0.534^1^ |
| **HB, Mean ± SD** | 145.72 ± 15.55 | 145.65 ± 15.71 | 145.88 ± 15.18 | 0.693^1^ |
| **PLT, Mean ± SD** | 204.93 ± 56.13 | 204.08 ± 55.22 | 206.92 ± 58.19 | 0.194^1^ |
| **Hct, Mean ± SD** | 43.78 ± 4.10 | 43.74 ± 4.15 | 43.88 ± 3.99 | 0.384^1^ |
| **TG, Mean ± SD** | 1.75 ± 1.45 | 1.77 ± 1.51 | 1.72 ± 1.29 | 0.375^1^ |
| **TC, Mean ± SD** | 5.10 ± 0.94 | 5.10 ± 0.94 | 5.09 ± 0.93 | 0.853^1^ |
| **HDL, Mean ± SD** | 1.44 ± 0.36 | 1.43 ± 0.36 | 1.44 ± 0.36 | 0.493^1^ |
| **LDL, Mean ± SD** | 3.22 ± 0.84 | 3.22 ± 0.84 | 3.22 ± 0.84 | 0.964^1^ |
| **Glucose, Mean ± SD** | 5.81 ± 1.57 | 5.83 ± 1.61 | 5.76 ± 1.50 | 0.297^1^ |
| **BUN, Mean ± SD** | 5.51 ± 1.41 | 5.53 ± 1.41 | 5.45 ± 1.40 | 0.161^1^ |
| **Creatinine, Mean ± SD** | 68.91 ± 16.19 | 68.92 ± 16.46 | 68.89 ± 15.55 | 0.966^1^ |
| **Uric acid, Mean ± SD** | 337.53 ± 87.86 | 337.21 ± 87.91 | 338.28 ± 87.77 | 0.750^1^ |
| **Monocyte, Mean ± SD** | 0.38 ± 0.15 | 0.38 ± 0.15 | 0.38 ± 0.14 | 0.554^1^ |
| **Neutrophil, Mean ± SD** | 3.45 ± 1.03 | 3.46 ± 1.05 | 3.43 ± 0.97 | 0.317^1^ |
| **Lymphocyte, Mean ± SD** | 1.96 ± 0.56 | 1.96 ± 0.56 | 1.95 ± 0.54 | 0.556^1^ |

Abbreviations: SD, standard deviation; CHD, coronary heart disease; BMI, body mass index; ALT, alanine aminotransferase; AST, aspartate aminotransferase; WBC, white blood cell count; RBC, red blood cell count; HB, hemoglobin; PLT, platelet count; Hct, hematocrit; TC, total cholesterol; TG, triglyceride; HDL, high-density lipoprotein; LDL, low-density lipoprotein; BUN, blood urea nitrogen.

**Table S2.** Results of Multivariate Logistic regression for Training Set.

| **Characteristic** | **N** | **Event N** | **OR** | **95% CI** | **p-value** |
| --- | --- | --- | --- | --- | --- |
| **Sex** |  |  |  |  |  |
| male | 1,160 | 180 | — | — |  |
| female | 1,134 | 43 | 0.02 | 0.01, 0.03 | **<0.001** |
| **Live alone** |  |  |  |  |  |
| No | 2,174 | 210 | — | — |  |
| Yes | 120 | 13 | 1.01 | 0.97, 1.04 | 0.314 |
| **Employment status** |  |  |  |  |  |
| No | 1,194 | 89 | — | — |  |
| Yes | 1,100 | 134 | 1.67 | 1.15, 2.44 | **0.007** |
| **Smoke** |  |  |  |  |  |
| No | 1,937 | 151 | — | — |  |
| Yes | 357 | 72 | 1.24 | 0.78, 1.95 | 0.357 |
| **Drink** |  |  |  |  |  |
| No | 1,732 | 128 | — | — |  |
| Yes | 562 | 95 | 1.21 | 0.88, 1.65 | 0.240 |
| **Calf circumference** | 2,294 | 223 | 0.89 | 0.83, 0.96 | **0.004** |
| **BMI** | 2,294 | 223 | 0.53 | 0.48, 0.58 | **<0.001** |
| **Systolic pressure** | 2,294 | 223 | 0.99 | 0.98, 1.00 | 0.159 |
| **Total bilirubin** | 2,294 | 223 | 0.95 | 0.92, 0.99 | **0.013** |
| **ALT** | 2,294 | 223 | 0.99 | 0.97, 1.00 | 0.097 |
| **HB** | 2,294 | 223 | 0.97 | 0.96, 0.99 | **<0.001** |
| **TC** | 2,294 | 223 | 1.50 | 1.23, 1.84 | **<0.001** |
| **Creatinine** | 2,294 | 223 | 0.96 | 0.94, 0.97 | **<0.001** |

**Abbreviations:** CI, confidence interval; OR, odds ratio; BMI, body mass index; ALT, alanine aminotransferase; HB, hemoglobin; TC, total cholesterol.


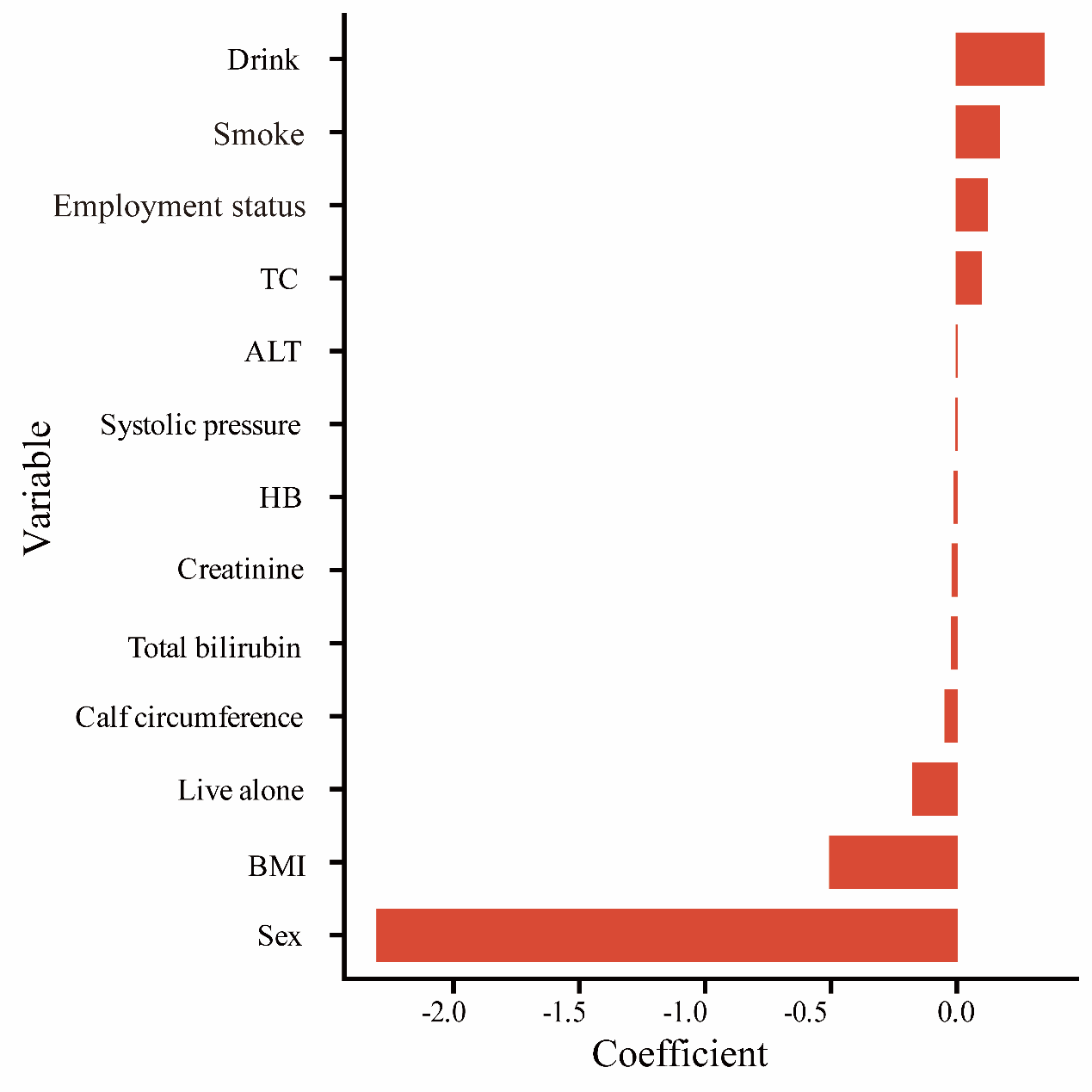


**Figure S1.** Predictor variables selected based on Lasso and their corresponding regression coefficients. Only display the variables with non-zero coefficients after Lasso screening.

Abbreviations: BMI, body mass index; ALT, alanine aminotransferase; HB, hemoglobin; TC, total cholesterol.


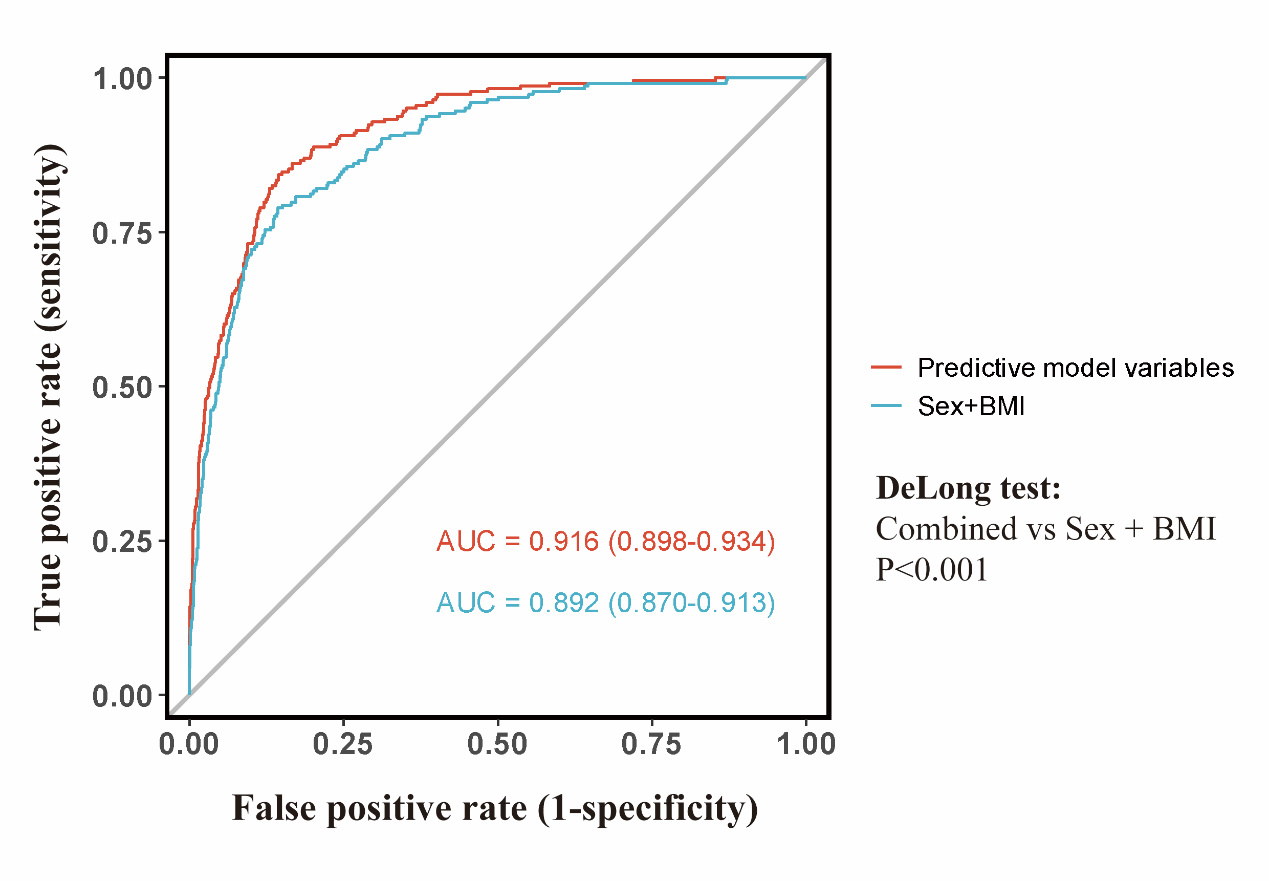


**Figure S2.** **Comparison of ROC Curves Between the Final Predictive Model and the Sex + BMI Model**
